# Curcumin attenuates LPS-induced inflammation in RAW 264.7 cells: A multifaceted study integrating network pharmacology, molecular docking, molecular dynamics simulation, and experimental validation

**DOI:** 10.1371/journal.pone.0335139

**Published:** 2025-10-23

**Authors:** Xiaojing Gong, Dingshan Xue, Hongyan Meng, Bing Xie, Lihua Zhao, Chuanhui Zang, Jingjing Kong

**Affiliations:** School of Medicine, Qingdao Binhai University, Qingdao, Shandong Province, P. R. China; Konkuk University, KOREA, REPUBLIC OF

## Abstract

**Background:**

Inflammation is a critical immune response that protects the body from infections and injuries. However, chronic inflammation can lead to diseases such as cancer. Curcumin, a bioactive compound extracted from *Curcuma longa*, has been widely studied for its anti-inflammatory properties. Despite extensive research, the comprehensive molecular mechanisms underlying curcumin’s anti-inflammatory effects, particularly its multi-target regulatory network, remain incompletely understood. This study aims to elucidate these mechanisms using an integrated approach combining network pharmacology, molecular docking, molecular dynamics simulation, and in vitro experimental validation.

**Methods:**

We utilized network pharmacology to identify potential targets and pathways involved in curcumin’s anti-inflammatory effects. Molecular docking and dynamics simulation were conducted to evaluate the binding affinity and stability of curcumin with key inflammatory targets. The anti-inflammatory effects of curcumin were further validated in vitro using LPS-induced RAW 264.7 cells. Cell viability, NO content, and mRNA expression levels of pro-inflammatory cytokines (*IL-1β*, *IL-6,* and *TNF*) were assessed.

**Results:**

Network pharmacology identified 135 potential targets for curcumin’s anti-inflammatory effects, with key pathways including TNF, HIF-1, PI3K-Akt, JAK-STAT, and MAPK signaling pathways. Molecular docking revealed strong binding affinities of curcumin with core targets such as IL-6, TNF, IL-1β, AKT1, and STAT3, with binding energies ranging from −6.2 to −7.5 kcal/mol. Molecular dynamics simulations demonstrated the stability of these complexes over a 100-nanosecond period. In vitro experiments showed that curcumin significantly reduced NO production and mRNA expression of *IL-1β*, *IL-6,* and *TNF* in LPS-induced RAW 264.7 cells, with optimal effects observed at a concentration of 125 μg/mL.

**Conclusion:**

Our study provides a comprehensive understanding of curcumin’s anti-inflammatory mechanisms through an integrated approach. The findings highlight curcumin’s potential as a therapeutic agent for inflammatory diseases. However, further in vivo studies are necessary to fully elucidate its therapeutic efficacy and mechanisms of action.

## 1. Introduction

Inflammation is a critical and intricate biological reaction of the body’s immune system, designed to protect against infections and injuries [1,2]. It is essential in the body’s defensive mechanisms, promoting the healing process and the restoration of tissue integrity [3–6]. Acute inflammation is a swift response that occurs immediately after an injury or infection, and it is beneficial as it aids in healing [7]. Conversely, when the body endures chronic, uncontrolled inflammation over the long term, the immune system may trigger an inflammatory response even in the absence of tissue damage, which can lead to the development of diseases, including cancer [8]. Therefore, it is crucial to manage inflammation effectively through well-designed therapeutic strategies.

Curcumin, a hydrophobic polyphenol, is extracted from the rhizome of Curcuma longa, which is also the main bioactive constituent of curcuminoids in turmeric, accounting for 77% of the content [9,10]. The pharmacological properties of curcumin have been extensively investigated, and it is typically characterized by its anticancer, anti-inflammatory, and antioxidant attributes [11–13]. Traditionally, curcumin has been employed as a coloring agent and anti-inflammatory agent in Indian and Chinese medicine [14]. Therefore, curcumin plays a significant role as an anti-inflammatory agent. Curcumin can reduce the production and release of several proinflammatory cytokines, such as IL-12, TNF, and IL-6 [15], and works by lowering the generation of ROS, restricting the influx of potassium, suppressing NF-κB, extracellularly regulated protein kinases (ERK) 1/2 and c-Jun N-terminal kinase (JNK), and preventing the activity of apoptosis-associated speck-like protein (ASC) [16]. However, while numerous studies have explored curcumin’s anti-inflammatory effects in disease-specific contexts, there remains a significant gap in understanding its overall anti-inflammatory mechanisms and integrated molecular pathways.

Network pharmacology utilizes public databases and past publications to predict the potential targets and pathways and offers a novel perspective for studying the pharmacological mechanisms involved in treating illnesses [17,18]. Additionally, network pharmacology allows researchers to examine diseases at a systemic level [19]. By constructing interactions between target proteins based on compounds and diseases and utilizing computer simulations to simulate the binding between compounds and their target proteins, network pharmacology can explain the mechanism of drug action [20]. Furthermore, Molecular docking, a virtual screening technique for the simulation of interactions between small molecular compounds and proteins, enzymes, or other biological molecules, is used to predict the binding modes between molecules and determine potential drug-active molecules by evaluating their affinity [4–6,21]. Molecular dynamics simulation (MDS) offers a comprehensive and systematic approach to simulating interactions and binding stability between small molecule monomers and protein targets [22]. Together, these methods are ideal for deconvoluting the poly-pharmacology of natural products like curcumin.

In this study, we employed an integrated strategy—combining network pharmacology, molecular docking, and molecular dynamics simulations—to systematically identify the overall anti-inflammatory targets and pathways of curcumin. Furthermore, we validated these predictions *in vitro* using an LPS-induced RAW 264.7 macrophage model, establishing a holistic mechanistic framework for curcumin’s bioactivity.

## 2. Materials and methods

### 2.1. Materials

Curcumin and lipopolysaccharides (LPS), were acquired from Shanghai Macklin Biochemical Technology Co., Ltd. (Shanghai, China). UNIQ-10 column total RNA purification kit and CCK8 (Cell Counting Kit-8) were purchased from Sangon Biotech Co., Ltd. (Shanghai, China). MagicSYBR Mixture and HiFiScript cDNA Synthesis Kit were sourced from Jiangsu Cowin Biotech Co., Ltd (Nanjing, China). The RAW 264.7 cell line and its media were obtained from Procell Life Science &Technology Co., Ltd. (Wuhan, China).

### 2.2. Target collection for curcumin and inflammation

SuperPred and Swiss Target Prediction were utilized to predict the targets associated with curcumin, based on its PubChem name (curcumin) and canonical SMILES string (COC1 = C(C = CC(=C1)/C = C/C(=O)CC(=O)/C = C/C2 = CC(=C(C = C2)O)OC)O) with the criteria of Probability≥50% for SuperPred and > 0.1 for Swiss Target Prediction. To identify inflammation-related targets, the GeneCards database was queried using the keyword “inflammation”. Additionally, the HERB database, a high-throughput experiment- and reference-guided repository of traditional Chinese medicine (http://herb.ac.cn/), was consulted to gather existing targets for both curcumin and inflammation. Any duplicate genes were removed. Subsequently, the targets for curcumin and those linked to inflammation were imported into the MyVenn tool within the Comparative Toxicogenomics Database (CTD) to identify the common targets that curcumin may influence in the context of inflammation.

### 2.3. GO and KEGG enrichment analysis

The common targets for the curcumin influencing inflammation were analyzed in the Database for Annotation, Visualization, and Integrated Discovery (DAVID, https://david.ncifcrf.gov/). for gene ontology (GO) annotation and kyoto encyclopaedia of genes and genomes (KEGG) pathway enrichment analyses. The graphs of GOTERM_BP_DIRECT, GOTERM_CC_DIRECT, and GOTERM_MF_DIRECT were downloaded to obtain GO enrichment analysis, while the graph of KEGG_PATHWAY was used for KEGG enrichment analysis. The target organism was *Homo sapiens*, the false discovery rate (FDR)-adjusted p-value was employed for elucidating the enrichment analysis, and a p-value < 0.01 was considered significant. The top ten items from enrich analyses were selected for visualization using the online platform https://www.bioinformatics.com.cn (last accessed on 10 Dec 2024), an online platform for data analysis and visualization.

### 2.4. Protein–protein interaction (PPI) network analysis

The protein–protein interaction (PPI) network of target genes was constructed using the Search Tool for the Retrieval of Interacting Genes/Proteins (STRING version 12.0, https://cn.string-db.org/), with the minimum required interaction score set to ≥0.7 [23]. Then the PPI network was graphically visualized and analyzed using the Cytoscape (version 3.9.1).

### 2.5. Molecular docking

The 2D (Two-dimensional) structure of the small molecule ligand was obtained through the PubChem database (http://pubchem.ncbi.nlm.nih.gov/), and then input into the Chem Office software to create its 3D (three-dimensional) structure. Subsequently, the protein targets with high-resolution crystal structures as molecular receptors were screened in AlphaFold DB (https://alphafold.ebi.ac.uk/). The selected proteins were processed using PyMOL software, including dehydration, deprotonation, and other preparatory steps. Molecular docking was performed using AutoDock Vina 1.5.6 software with a docking box of 30 × 30 × 30 Å centered on the active site (coordinates from AlphaFold DB) and a grid spacing of 0.375 Å. The docking procedure was validated via re-docking, yielding an RMSD <2 Å for reference ligands, confirming reliability. To explore the interaction between the protein receptors and the small molecule ligand, the interactions between the test compounds and key residues within the protein targets were visualized and analyzed using Discovery Studio 2019 and PyMOL software. 2D and 3D interaction diagrams were generated to provide detailed insights into the binding interactions, including hydrogen bonds, hydrophobic interactions, and other relevant contacts.

### 2.6. Molecular dynamics

A 100-nanosecond (ns) molecular dynamics simulation of the complex was executed using GROMACS 2023. The protein was parameterized with the CHARMM36 force field (chosen for its accuracy in modeling protein backbone dynamics), whereas the ligand topology was generated using the GAFF2 force field (optimized for curcumin’s polyphenolic structure with enhanced hydrogen bond parameters). Curcumin was parameterized via antechamber in AmberTools, generating partial charges using the RESP method and validating against experimental bond lengths. Periodic boundary conditions were implemented, and the protein-ligand complex was placed within a cubic simulation box. This box was solvated using the TIP3P water model. Electrostatic interactions were treated using the Particle Mesh Ewald (PME) method, with the Verlet cutoff scheme employed. The system underwent equilibration under both isothermal-isochoric (NVT) and isothermal-isobaric (NPT) ensembles for 100,000 steps, featuring a coupling constant of 0.1 picoseconds (ps) and a duration of 100 ps. Both van der Waals and Coulomb interactions were computed with a cutoff distance of 1.0 nanometer (nm). Ultimately, the MD simulation was conducted at a constant temperature of 300 Kelvin (K) and a constant pressure of 1 bar for a total span of 100 ns using GROMACS 2023.

### 2.7. Experiment validation

#### 2.7.1. Cell culture.

RAW 264.7 cells were cultured in the DMEM supplemented with 10% FBS (Fetal Bovine Serum) and 1% Penicillin Streptomycin Solution at 37°C and 5% CO_2_. Cell passages were conducted at intervals of 3–4 days, with a 24-hour recovery period allowed for the cells prior to any treatment.

#### 2.7.2. Cell viability.

RAW 264.7 cells were seeded at a density of 5000 cells per well in a 96-well plate and incubated overnight. Subsequently, for a duration of 24 hours, the cells were exposed to various concentrations of Curcumin, ranging from 10 μg/mL to 500 μg/mL, in the presence of LPS (1 μg/mL). A model group was established where cells were solely treated with LPS (1 μg/mL), while a blank group received no treatment at all. For viability assessment, CCK-8 solution was added into each well, and the cells were incubated at 37 °C for an hour. The optical density (OD) was then measured using the Spark Multimode Reader Platform (Tecan, Switzerland) at a wavelength of 450 nm. The cell survival rate was calculated as follows: Cell viability (%) = [(OD_Sample_ – OD_Blank_)/ (OD_Control_ – OD_Blank_)] × 100%.

#### 2.7.3. Determination of NO content.

The concentration of NO was determined using the Griess reagent. Briefly, 100 μL of cell lysis supernatant was mixed with an equal volume of the Griess reagent in 96 – well flat – bottom plate. The Griess reagent consisted of 0.1% N – [1 – naphthyl] ethylenediamine dihydrochloride (in distilled water) and 1% sulfanilamide (in 5% phosphoric acid). After a 10-minute incubation, the absorbance was measured at 550 nm. NO content was calculated based on a sodium nitrite standard curve.

#### 2.7.4. Measurement of proinflammatory cytokine and target gene.

The cellular total RNA was extracted using the UNIQ-10 column total RNA purification kit, according to the manufacturer’s protocol. cDNA was synthesized using the HiFiScript cDNA synthesis kit. qRT-PCR was performed on a CFX Connect Real-Time PCR System (Bio-Rad, USA) in a total reaction volume of 20 μL, containing 10 μL of 2 × Magic SYBR Mixture, 4 μL of each primer (2 μM), and 2 μL of cDNA. Relative expression levels of proinflammatory cytokines and target genes were calculated using the 2^−ΔΔCq^ method, with β-actin as the endogenous control. Primer sequences are listed in Table 1.

**Table 1:** Primers used for qRT-PCR.

Primer	Sequence
TNF-F	5’-TGATCGGTCCCCAAAGGGATG-3’
TNF-R	5’-TTGGTGGTTTGCTACGACGTGG-3’
IL-1β-F	5’-GCAACTGTTCCTGAACTCAACT-3’
IL-1β-R	5’-ATCTTTTGGGGTCCGTCAACT-3’
IL-6-F	5’-TGATGCACTTGCAGAAAACAATCTGA-3’
IL-6-R	5’-AGCTATGGTACTCCAGAAGACCAGAGG-3’
β-actin-F	5’-GGCTGTATTCCCCTCCATCG-3’
β-actin-R	5’-CCAGTTGGTAACAATGCCATGT-3’

### 2.8. Statistical analysis

All experiments were performed in quadruplicate, and data were presented as the mean ± standard error of the mean (SEM). Statistical analyses were conducted using GraphPad Prism software (version 9.0). One-way ANOVA was used to compare groups, followed by Duncan’s multiple range test. A p-value < 0.05 was considered statistically significant.

## 3. Results

### 3.1. Analysis of curcumin and inflammation targets

**Fig 1:**
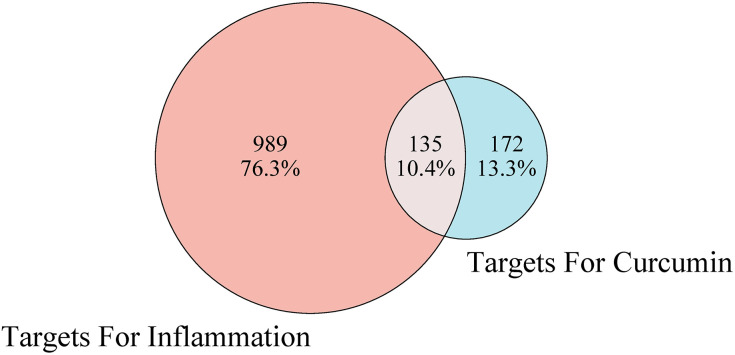
Venn diagram of overlapping target genes between curcumin and inflammation.

The structure of curcumin was obtained from PubChem. There were 307 curcumin related target genes by SuperPred, Swiss Target Prediction and HERB. From the GeneCards database, 949 inflammation related targets were identified among 11,367 entries of protein coding with a relevance score >5.0 while 433 inflammation related targets from HERB. After removing duplicates, 1,124 inflammation-related genes were identified for further analysis. Matching inflammation-related genes with the curcumin targets, 135 genes were selected as potential targets for the anti-inflammatory effect of curcumin (Fig 1).

### 3.2. Enrichment analysis

**Fig 2:**
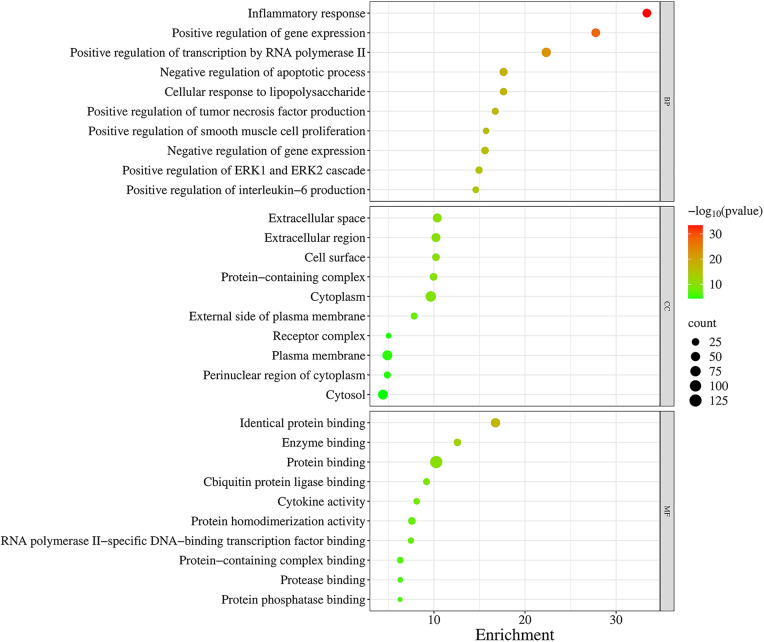
Bubble diagram for gene ontology function enrichment analysis. Top 10 biological processes (BP), cellular component (CC), and molecular function (MF) were sorted based on the FDR.

After GO functional enrichment analysis by DAVID, we obtained 341 GO-enriched terms (*P* < 0.01), including 272 terms in the BP domain, 22 terms in the CC domain, and 47 terms in the MF domain. Each category’s top 10 GO entries were sorted based on the FDR (Fig 2): The main enriched BP were related to inflammatory response and positive regulation of gene expression, the main enriched CC were extracellular space, extracellular region and cell surface, and the MF were identical protein binding, enzyme binding and protein binding.

**Fig 3:**
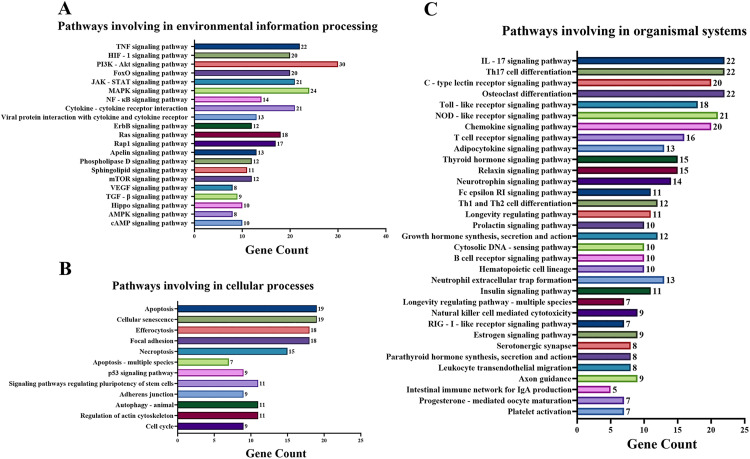
Classified enriched KEGG pathways of curcumin targets against inflammation. **(A)** Pathways involving in environmental information processing. **(A)** Pathways involving in cellular processes. **(C)** Pathways involving in organismal systems.

**Fig 4:**
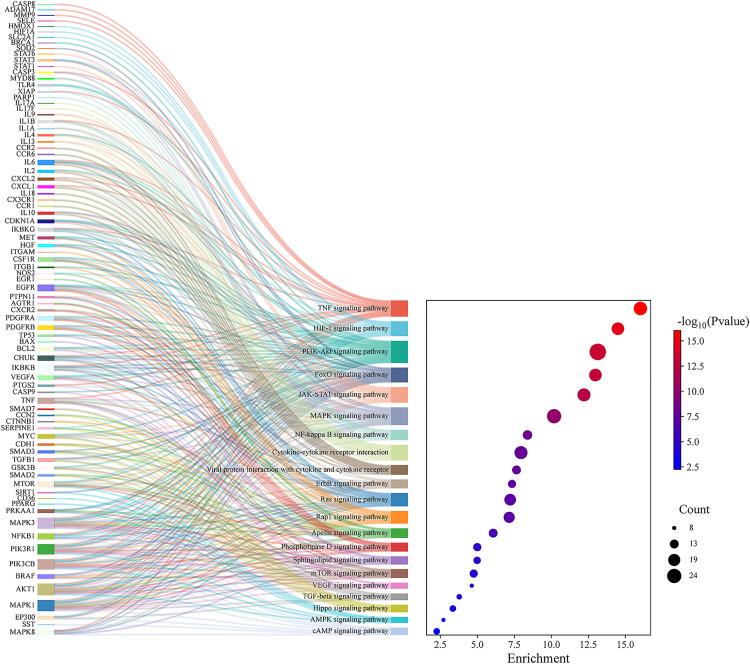
Pathway enrichment analysis for the targets of curcumin against inflammation.

KEGG pathway enrichment analysis identified 139 significant pathways with FDR < 0.01. These pathways were further classified into four major groups: 21 pathways of environmental information processing (Fig 3A), 12 pathways of cellular processes (Fig 3B), 35 pathways of organismal systems (Fig 3C), and 71 pathways were related to human disease S1 Fig). Inflammation is usually a critical biological reaction of the body’s immune system to stimulus, so here the pathways for the environmental information processing were selected for the Sankey and dot plot chart: the TNF signaling pathway, HIF-1 signaling pathway, PI3K-Akt signaling pathway, JAK-STAT signaling pathway, MAPK signaling pathway, NF-kappa B signaling pathway and Cytokine-cytokine receptor interaction were the main signaling pathways associated with inflammation (Fig 4).

### 3.3. PPI network construction and analysis

**Fig 5:**
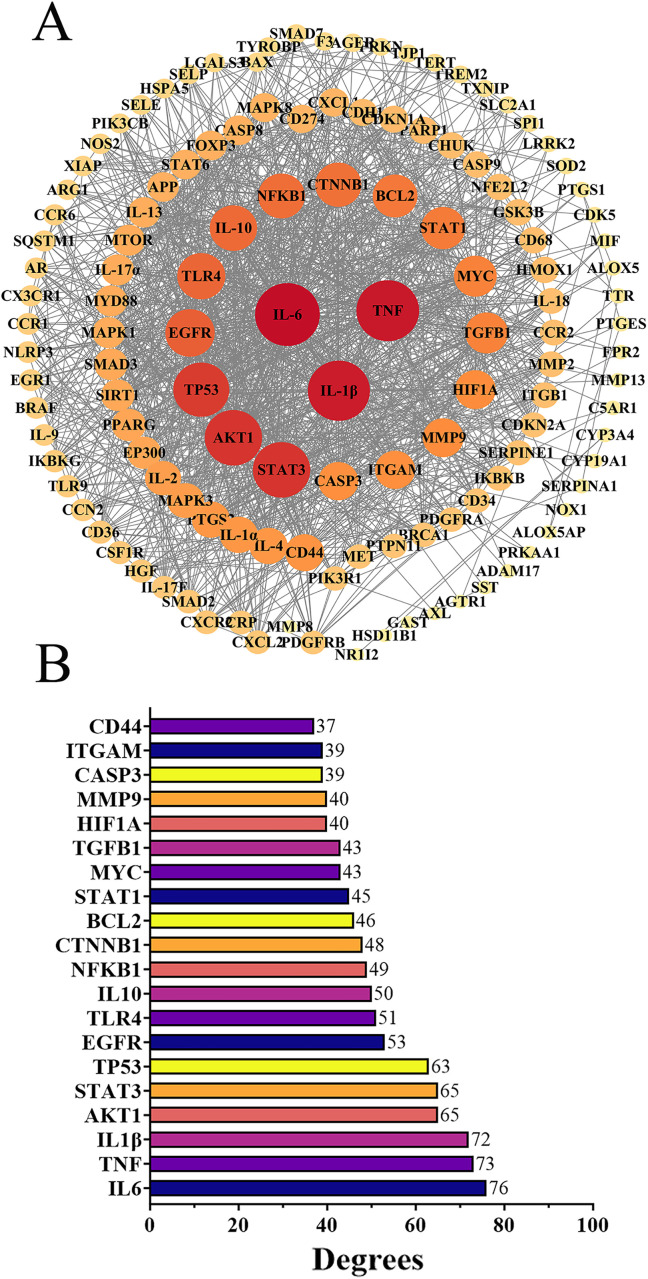
Comprehensive PPI Network Analysis. **(A)** Cytoscape-Reconstructed PPI Network. Visual Representation with Node Colors and Sizes Proportional to Target Degree. **(B)** Top 20 Targets in PPI Network Based on Degree Values.

A total of 135 common targets were imported into the STRING database to generate the PPI network. Subsequently, Cytoscape version 3.9.1 was utilized to reconstruct and analyze the function-related PPI network. Fig 5A depicts the PPI network, comprising 1336 interactions (edges) among 130 nodes. The average node degree, which represents the average number of interactions per node, was 20.554. The edges within the network signify the interactions between the nodes. The top 20 targets with a degree value of 31 or higher are presented in Fig 5B. By employing the “CytoNCA” plugin within Cytoscape 3.9 software, we identified the top 10 core targets: IL-6, TNF, IL-1β, AKT1, STAT3, TP53, NFKB1, EGFR, IL-10, and STAT1.

### 3.4. Molecular docking

**Fig 6:**
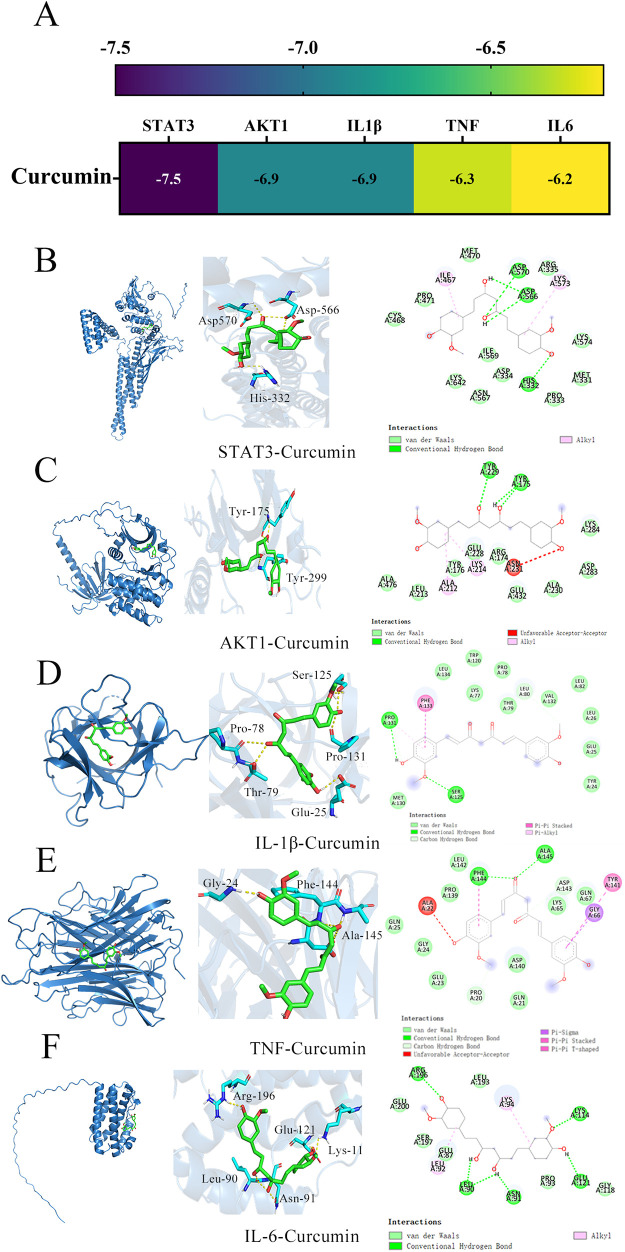
The molecular docking results between curcumin and the core targets. **(A)** The heatmap for affinity of curcumin to key targets. **(B)** Curcumin-STAT3. **(C)** Curcumin-AKT1. **(D)** Curcumin-TNF. **(E)** Curcumin-IL-1β. **(F)** Curcumin-IL-6.

In order to verify the binding activity of curcumin to the targets, core targets (IL-6, TNF, IL-1β, AKT1 and STAT3) were subjected to molecular docking. The affinity of curcumin to key targets are shown in Fig 6A. The results showed that the binding energies of IL-6, TNF, IL-1β, AKT1 and STAT3 were −6.2, −6.5, −6.5, −6.9 and −7.5 kcal/mol respectively. Our results show that the binding energies of curcumin to the target are all lower than −5.0 kcal/mol and have good affinity. Finally, visual analysis is carried out by Pymol 2.0 software (Fig 6B-F).

### 3.5. Molecular dynamics stimulation

**Fig 7:**
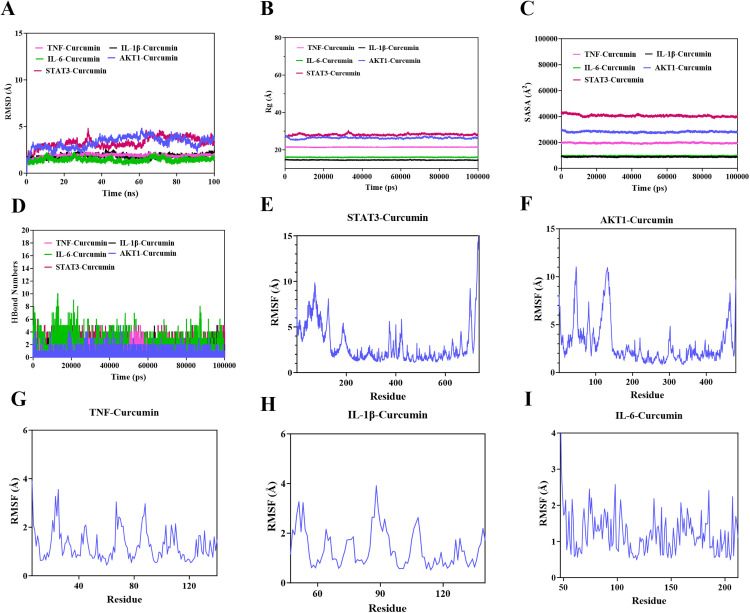
The Molecular dynamics stimulation validation parameter of protein/ligand models included (A) the RMSD profile, (B) the Rg descriptor, (C) SASA value, (D) the hydrogen bonds (H-bonds) numbers and (E-I) the RMSF value, derived from MD trajectories.

To further demonstrate the binding of curcumin to the core targets, the simulations were performed, and the root mean square deviation (RMSD), the radius of gyration (Rg), solvent accessible surface area (SASA), hydrogen bonds(H-bonds), and root mean square fluctuation (RMSF) of the curcumin-targets (IL-6, TNF, IL-1β, AKT1 and STAT3) complexes were calculated (Fig 7). The root mean square deviation (RMSD) serves as a reliable metric for assessing the conformational stability of proteins and ligands, as well as quantifying the deviation between atomic positions and their initial states. A smaller deviation indicates enhanced conformational stability. Consequently, RMSD was utilized to evaluate the equilibrium state of the simulation system. As IL-lustrated in Fig 7A, the TNF-Curcumin complex system attained equilibrium after 98 nanoseconds (ns), fluctuating around 24.1 Å; Similarly, the IL-1β-Curcumin complex system reached equilibrium after 55 ns, with fluctuations centering around 10 Å; The STAT3-Curcumin complex system equilibrated after 98 ns, fluctuating around 8.1 Å; Notably, the IL-6-Curcumin complex system achieved equilibrium after just 10 ns, with fluctuations near 1.7 Å, indicating a remarkably low RMSD value. Further examination revealed slight fluctuations in the radius of gyration (Rg) and solvent accessible surface area (SASA) of the TNF-Curcumin, IL-1β-Curcumin, STAT3-Curcumin, IL-6-Curcumin, and AKT1-Curcumin complex systems during movement, suggesting conformational changes as depicted in Fig 7 B and C.

Hydrogen bonds play a pivotal role in the binding of ligands to proteins. Fig 7D displays the number of hydrogen bonds formed between small molecules and target proteins throughout the kinetic process. Specifically, the TNF-Curcumin complex system exhibits a range of 0–7 hydrogen bonds, with an average of approximately 3 hydrogen bonds in most cases. The IL-1β-Curcumin complex system ranges from 0 to 5 hydrogen bonds, averaging around 2 in most instances. The STAT3-Curcumin complex system has a range of 0–6 hydrogen bonds, with an average of approximately 4. The IL-6-Curcumin complex system ranges from 0 to 10 hydrogen bonds, averaging approximately 4 in most cases. Lastly, the AKT1-Curcumin complex system has a range of 0–5 hydrogen bonds, averaging around 2. These findings indicate robust hydrogen bonding interactions within the TNF-Curcumin, IL-1β-Curcumin, STAT3-Curcumin, IL-6-Curcumin, and AKT1-Curcumin complex systems.

The root mean square fluctuation (RMSF) is indicative of the flexibility of amino acid residues within proteins. As depicted in Fig 7E-I, the RMSF values of the TNF-Curcumin, IL-1β-Curcumin, STAT3-Curcumin, IL-6-Curcumin, and AKT1-Curcumin complex systems are relatively low, mostly below 10 Å, suggesting limited flexibility and enhanced stability.

Finally, using the binding conformation of the complex, the binding free energy of the small molecule with the target protein was calculated using the MM/PBSA method. The binding free energies of the TNF-Curcumin, IL-1β-Curcumin, STAT3-Curcumin, IL-6-Curcumin, and AKT1-Curcumin complex systems are −57.341 kJ/mol, −119.612 kJ/mol, −94.543 kJ/mol, −23.770 kJ/mol, and −140.328 kJ/mol, respectively. All the results confirmed the observation that all ligands were able to bind to Curcumin with an appreciable binding affinity.

### 3.6. The influence of curcumin on LPS-Induced RAW 264.7 cell viability

**Fig 8:**
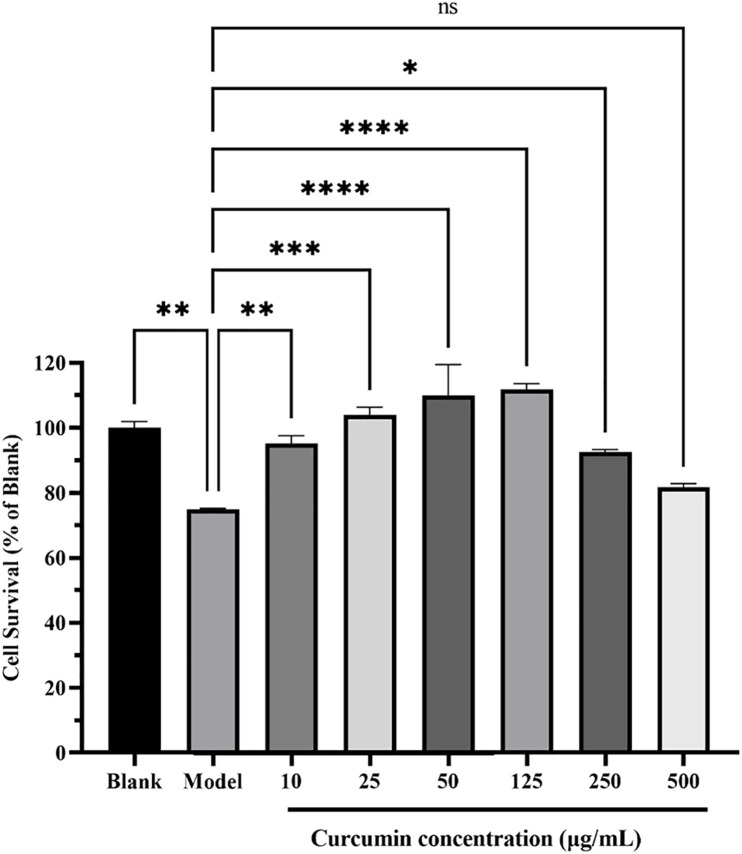
The effect of curcumin on LPS-Induced RAW 264.7 cell viability. Data are presented as mean ± SEM (n = 4). Data are presented as mean ± SEM (n = 4). Statistical significance was determined by one-way ANOVA with Duncan’s post-hoc test: ns (p > 0.05), *p < 0.05, **p < 0.01, ***p < 0.001, ****p < 0.0001 vs. LPS model group. Analyses and visualization performed using GraphPad Prism 9.0.

Compared to the blank group (100% cell viability), LPS treatment significantly reduced the viability of RAW 264.7 cells to 74.87% (p < 0.01). In contrast, curcumin treatment exhibited a dose-dependent enhancement of cell viability. Specifically, at curcumin concentrations of 10, 25, 50, and 125 μg/mL, cell viability improved markedly compared to the model group, with statistical significance reaching p < 0.001 for 10 and 25 μg/mL, and p < 0.0001 for 50 and 125 μg/mL. The peak cell viability was observed at 125 μg/mL, where it reached 111% of the blank group’s viability; subsequently, cell vitality exhibits a declining trend with the increase in treatment concentration and at a concentration of 500 μg/mL, cell viability diminishes to a level that shows no statistically significant difference compared to the model group (Fig 8).

### 3.7. The curcumin anti-inflammatory impact on LPS-induced RAW 264.7 cells

**Fig 9:**
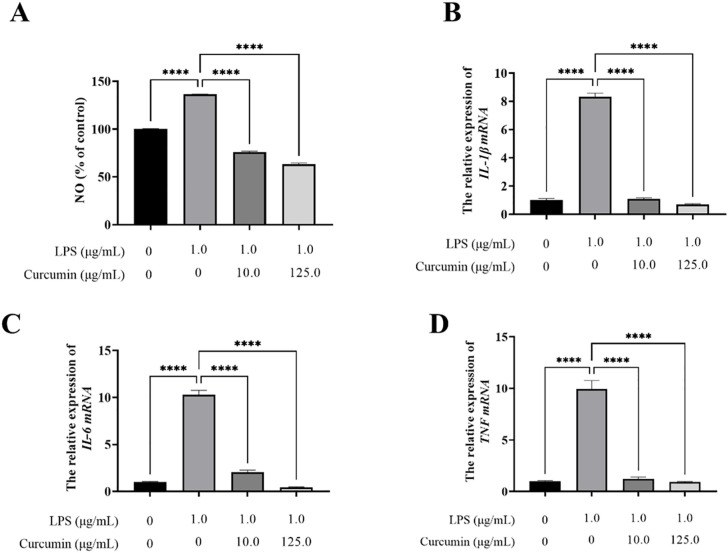
The Impact of curcumin on the Levels of (A) Nitric Oxide (NO), (B) IL-1β mRNA, (C) IL-6 mRNA, and (D) TNF mRNA. Data are presented as mean ± SEM (n = 4). ****p < 0.0001 vs. LPS group (one-way ANOVA with Duncan’s multiple range test, analyzed and visualization using GraphPad Prism 9.0).

Compared to the control group (set at 100% baseline), LPS treatment (1.0 μg/mL) induced a significant inflammatory response, elevating nitric oxide (NO) production to 136.25% (p < 0.0001) and markedly upregulating mRNA expression of pro-inflammatory cytokines: IL-1β (8.32-fold increase, p < 0.0001), IL-6 (10.27-fold increase, p < 0.0001), and TNF (9.95-fold increase, p < 0.0001); Curcumin intervention exhibited dose-dependent anti-inflammatory effects, effectively reversing these LPS-induced alterations. At 10 μg/mL, curcumin reduced NO levels to 75.82% (p < 0.0001 vs LPS) and suppressed cytokine expression by 87.02% (IL-1β), 80.04% (IL-6), and 87.63% (TNF) (all p < 0.0001 vs LPS). The 125 μg/mL dose demonstrated superior efficacy, lowering NO to 63.02% (p < 0.0001 vs LPS) and further decreasing cytokine levels by 91.59% (IL-1β), 95.67% (IL-6), and 90.74% (TNF) (all p < 0.0001 vs LPS) (Fig 9). Notably, network pharmacology analysis had previously identified IL-1β, IL-6, and TNF as key targets of curcumin, and these experimental findings confirm dose-dependent modulation of these cytokines, thereby validating the mechanistic predictions.

## 4. Discussion

Inflammation **i**s a crucial and essential immune response in the body, playing a pivotal role in eliminating harmful irritants and promoting the repair of damaged tissues following exposure to injurious stimuli [24]. During the initiation of an inflammatory response, the body orchestrates the production of a complex cascade of inflammatory cells and mediators. However, when these inflammatory factors become dysregulated, they may lead to tissue damage or even life-threatening consequences [25]. Moreover, the therapeutic targeting of inflammation is highly challenging, given its complex involvement of a multitude of inflammatory mediators and pathways, leading to a broad spectrum of pathological changes. Currently, inflammation treatment primarily focuses on inhibiting cyclooxygenase and suppressing various mediators produced during the inflammatory response. This approach, however, can easily induce adverse effects on the body, including peptic ulcers, abnormal liver and kidney function, and cardiovascular and cerebrovascular diseases [26]. Herbs have been widely utilized in the treatment of inflammation-related diseases due to their unique characteristics of containing multi-compounds, targeting multiple pathways, and employing multiple mechanisms to alleviate inflammation [27]. *Curcuma longa*, commonly known as turmeric, has been utilized **a**s an anti-inflammatory agent in both Chinese and Indian medicinal practices for an extended period [28]. Curcumin, the primary bioactive constituent of the curcuminoids present in turmeric, exhibits notable anti-inflammatory properties. NO serves as a pivotal inflammatory mediator in host defense mechanisms: it is primarily produced by activated macrophages, acts as a key cytotoxic/cytostatic effector molecule in innate immune responses and diverse physiological/pathological scenarios, and its production is frequently inhibited by the anti-inflammatory activity of numerous bioactive substances [6]. In the present study, we systematically evaluated the anti-inflammatory efficacy of curcumin using an in vitro model of LPS-induced inflammation in RAW 264.7 murine macrophage cells. Our experimental results revealed that curcumin treatment led to a statistically significant (p < 0.05) attenuation of NO production in LPS-activated macrophages (Fig. 8A), indicating its capacity to regulate macrophage-mediated inflammatory cascades. Moreover, curcumin treatment could suppress the secretion of pro-inflammatory cytokine IL-1β, IL-6, and TNF (Fig. 8B-D).

Through network pharmacology analysis, we systematically identified curcumin-associated therapeutic targets by integrating computational predictions and disease-related databases. Using SuperPred, Swiss Target Prediction, and HERB platforms, we retrieved 307 genes potentially targeted by curcumin. Concurrently, 1124 inflammation-related genes were curated from Genecards and HERB databases through keyword-based screening. By performing intersection analysis of these two datasets, we pinpointed 135 genes as core nodes bridging curcumin’s pharmacodynamic profile to anti-inflammatory mechanisms. Further GO enrichment analysis revealed that these targets were intimately linked to various biological processes, including inflammatory response, positive regulation of gene expression and transcription from RNA polymerase II promoters, negative regulation of the apoptotic process, cellular response to lipopolysaccharide, and positive regulation of tumor necrosis factor production, among many others—the majority of which were directly related to inflammation. Additionally, KEGG enrichment analysis demonstrated that the TNF signaling pathway, HIF1 signaling pathway, PI3K-Akt signaling pathway, JAK-STAT signaling pathway, and MAPK signaling pathway were pivotal pathways within the category of environmental information processing. TNF signaling pathway plays a central role in orchestrating mammalian inflammatory responses, which promotes inflammation either directly by inducing inflammatory gene expression or indirectly by triggering cell death [29]. HIF-mediated hypoxia signaling pathway is therefore not only crucial for coordinating cellular responses to low oxygen tension but also for immune responses and inflammation [30]. The activity of the PI3K/Akt pathway is typically suppressed when exposed to inflammatory stimuli, and activation of the PI3K/Akt signaling cascade resulted in decreased protein expression levels of key inflammatory mediators such as COX2, TNFα, and IL-6 [31]. JAK – STAT signaling pathway is an important hub of cytokine signaling and is crucial for the regulation of immune and inflammatory responses [32]. MAPK signaling pathway is a classic immune inflammation-related signaling pathway and the core of the regulation of immune inflammatory response and can be activated by oxidative stress and cytokines including TNF-α [33]. These signaling pathways are closely related to inflammatory response. Notably, our pathway enrichment analysis is largely consistent with the findings of other network pharmacology studies on curcumin [34–36]. However, we have expanded upon these previous studies by primarily focusing on the enrichment of curcumin’s impact on pathways involved in environmental processes. Additionally, we have refined the signaling pathways related to inflammatory effects and excluded pathways associated with human diseases, thereby presenting a more intuitive visualization of the anti-inflammatory pathways of curcumin. Our study, provides a more comprehensive view by identifying multiple signaling pathways (TNF, HIF1, PI3K-Akt, JAK-STAT, and MAPK) through which curcumin exerts its effects.

**Fig 10:**
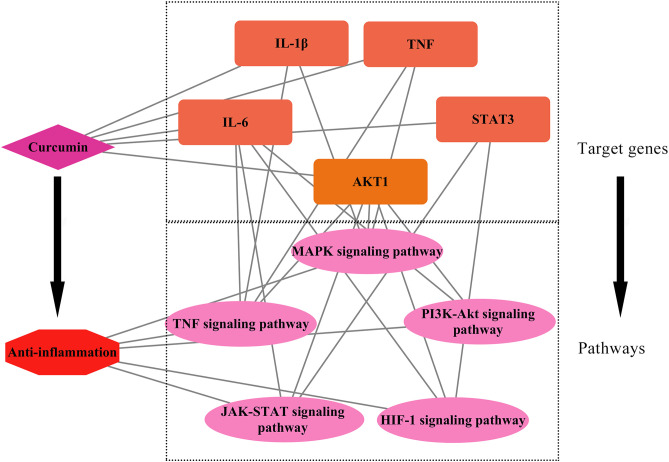
Schematic representation of curcumin’s anti-inflammatory mechanism: modulation of key hub target genes and enriched signaling pathways.

PPI analysis was conducted to elucidate the interaction networks among potential targets mediating curcumin’s anti-inflammatory effects. Through network topology evaluation, five hub genes—IL-6, TNF, IL-1β, AKT1, and STAT3—were identified based on their significantly higher degrees compared to other nodes. These hub genes were prioritized for subsequent molecular docking and dynamics studies. Molecular docking analyses revealed that curcumin exhibits favorable binding affinity across all five targets, with STAT3 showing the strongest interaction (−7.5 kcal/mol), followed by AKT1 (−6.9 kcal/mol) (Fig. 6). These results align with cross-study validations: despite minor variations in binding energies across docking tools (e.g., AutoDock Vina, Glide, MM/GBSA), the consistent trend of negative binding energies across all targets—particularly STAT3 and AKT1—supports curcumin’s multitarget potential [37,38]. Comparative analyses with quercetin further highlighted curcumin’s superior STAT3 binding (higher affinity) but weaker AKT1 binding, suggesting target-specific ligand optimization opportunities [39]. Molecular dynamics simulations provided temporal insights into binding stability. RMSD analysis showed that IL-6-curcumin, IL-1β-curcumin, and TNF-curcumin complexes reached equilibrium at 10 ns, 55 ns, and 98 ns, respectively—faster than STAT3-curcumin and AKT1-curcumin systems (Fig. 7A). This kinetic profile suggests that curcumin may first intercept soluble inflammatory factors (e.g., IL-6, TNF) to block pro-inflammatory signaling (e.g., TNF pathway), followed by sustained engagement with intracellular targets like STAT3/AKT1 to modulate downstream inflammatory cascades. Binding free energy calculations via MM/PBSA confirmed high stability for curcumin-AKT1/STAT3 complexes, consistent with their roles in signaling pathways. These molecular-level findings not only corroborate existing literature on curcumin’s anti-inflammatory actions but also reveal a novel STAT3-dependent regulatory mechanism for cytokine signaling. The strong STAT3 binding affinity, in particular, warrants further investigation into its potential for precision therapeutic targeting. The schematic (Fig 10) illustrates that curcumin alleviates inflammation by modulating enriched signaling pathways via key hub target genes.

Compared with the published paper focusing on disease – specific contexts like Parkinson’s disease (via PI3K/AKT activation) [34], osteoarthritis (via p38MAPK inhibition) [35], or skin inflammation (via NF-κB and MAPK pathways) [36], our study centers on fundamental anti – inflammatory mechanisms. While those studies validated curcumin’s efficacy in specific diseases, our work, through network pharmacology, molecular docking, and in vitro experiments, goes beyond confirming well – known effects on TNF and IL – 6. We identify STAT3 as a critical validated hub, which is rarely emphasized in prior network pharmacology studies on curcumin. Moreover, we reveal curcumin’s modulation of multiple interconnected pathways (TNF - HIF-1 - PI3K-Akt – JAK-STAT), unlike previous research that often focused on isolated pathways. This multi-pathway crosstalk insight, along with the validation of STAT3 as a core target, provides unique mechanistic understanding, showing how curcumin simultaneously targets multiple nodes in the inflammatory network to exert its effects, a perspective less explored in disease-specific curcumin studies.

Furthermore, the NO inhibitory activity of curcumin at 125 μg/mL achieved 53.75% suppression, comparable to polyphenolic agents such as fisetin, quercetin, cranberry polyphenol extracts, and herbal compounds, though only half the efficacy of single-target pharmaceuticals [40–42]. Clinically, curcumin’s multi-target mechanism—simultaneously modulating TNF, IL-6, IL-1β, STAT3, and AKT1 pathways—contrasts sharply with single-target agents like COX-2 inhibitors (e.g., celecoxib) or TNF inhibitors (e.g., infliximab). Unlike COX-2 inhibitors, which primarily reduce prostaglandin synthesis but carry cardiovascular risks, or TNF inhibitors that suppress one cytokine while potentially triggering compensatory IL-6/STAT3 activation, curcumin’s broad-spectrum action may circumvent these limitations. By simultaneously engaging multiple pathways, it reduces pathway redundancy and delays resistance development—a critical advantage in poly-cytokine-driven diseases such as rheumatoid arthritis or inflammatory bowel disease. However, this polypharmacology necessitates careful risk assessment. Potential concerns include non-specific immunosuppression or pharmacokinetic interactions with immunosuppressants (e.g., corticosteroids), requiring precise dosing optimization. Preclinically, curcumin exhibits synergy with existing therapies: it may enhance TNF inhibitor efficacy by blocking IL-6/STAT3 compensatory feedback, while its AKT1-modulating effects could counteract COX-2 inhibitor-induced apoptosis resistance. Such combinations demand rigorous preclinical validation to balance efficacy and safety.

A persistent translational challenge remains curcumin’s poor bioavailability. The in vitro effective concentration (125 μg/mL) exceeds clinically achievable plasma levels (nanomolar range) by orders of magnitude. To reconcile in vitro efficacy with in vivo feasibility, systematic bioavailability-enhancement strategies are therefore required. Promising approaches include: (1) polymeric nanoparticle platforms (e.g., PLGA-encapsulated formulations) that increase apparent solubility, protect curcumin from hydrolytic degradation, and facilitate receptor-mediated cellular uptake; (2) liposomal carriers that improve both circulatory half-life and tissue-specific distribution; and (3) co-administration of the UDP-glucuronosyltransferase inhibitor piperine, which has been shown in pre-clinical models to elevate systemic curcumin exposure by up to 20-fold without overt toxicity. Addressing this hurdle through targeted delivery systems and pharmacokinetic optimization remains essential to translate preclinical promise into clinical utility.

While this study provides valuable insights into curcumin’s anti-inflammatory mechanisms through an integrated computational and experimental approach, several limitations should be acknowledged. The exclusive reliance on RAW 264.7 macrophage cells for in vitro validation may not adequately capture the complexity of inflammatory responses in human physiological systems or animal models. Molecular docking and dynamics simulations were conducted using predicted or homology-modeled protein structures rather than experimentally determined crystal structures, potentially impacting the reliability of binding affinity and stability assessments. The investigation focused on a restricted panel of inflammatory markers (IL-1β, IL-6, TNF, and NO), omitting other potentially relevant cytokines and pathways. Most significantly, the absence of in vivo validation substantially limits the translational potential of these findings. To address the translational gap, future in vivo studies should prioritize specific disease models that mirror human inflammatory pathologies. For rheumatoid arthritis, the collagen-induced arthritis (CIA) model in mice or rats would allow direct evaluation of curcumin’s impact on joint inflammation, synovial hyperplasia, and serum cytokine profiles (e.g., IL-6, TNF, and STAT3 phosphorylation). In inflammatory bowel disease, the dextran sulfate sodium (DSS)-induced colitis model enables assessment of curcumin’s effects on mucosal barrier integrity, neutrophil infiltration, and pro-inflammatory cytokine expression (IL-1β, IL-6) in colonic tissue. For cardiovascular inflammation, the ApoE ⁻ / ⁻ mouse model of atherosclerosis provides a platform to test curcumin’s ability to suppress vascular inflammation, macrophage accumulation, and foam cell formation via AKT1/STAT3 modulation. Additionally, the cecal ligation and puncture (CLP) sepsis model would validate curcumin’s systemic anti-inflammatory potential in a life-threatening inflammatory condition. These models, combined with pharmacokinetic/pharmacodynamic (PK/PD) analyses to correlate in vivo efficacy with curcumin exposure levels achieved through bioavailability-enhanced formulations, would rigorously bridge preclinical findings to clinical applications.

## 5. Conclusions

In conclusion, our study provides a comprehensive investigation into the anti-inflammatory mechanisms of curcumin using an integrated approach that combines network pharmacology, molecular docking, molecular dynamics simulation, and in vitro experimental validation. Through network pharmacology, we identified 135 potential targets for curcumin’s anti-inflammatory effects and revealed key signaling pathways, including TNF, HIF-1, PI3K-Akt, JAK-STAT, and MAPK pathways. Molecular docking and dynamics simulations demonstrated that curcumin exhibits strong binding affinity and stability with core inflammatory targets such as IL-6, TNF, IL-1β, AKT1, and STAT3. In vitro experiments using LPS-induced RAW 264.7 cells further validated curcumin’s ability to significantly reduce the production of pro-inflammatory cytokines (IL-1β, IL-6, and TNF) and NO, thereby mitigating inflammation. Although our findings highlight the promising anti-inflammatory potential of curcumin, further in vivo studies are necessary to fully elucidate its mechanisms and therapeutic efficacy in inflammatory diseases.

## Supporting information

S1 Table The overlap genes list for Fig 1. (XLSX)

S1 Fig Classified enriched KEGG pathways of curcumin targets against inflammation involving in human disease. (TIF)
