# Supplementary figures and images for: Curcumin attenuates LPS-induced inflammation in RAW 264.7 cells: A multifaceted study integrating network pharmacology, molecular docking, molecular dynamics simulation, and experimental validation
Source: PLoS One. 2025 Oct 23;20(10):e0335139. doi: 10.1371/journal.pone.0335139 (PMC12548870; doi:10.1371/journal.pone.0335139)

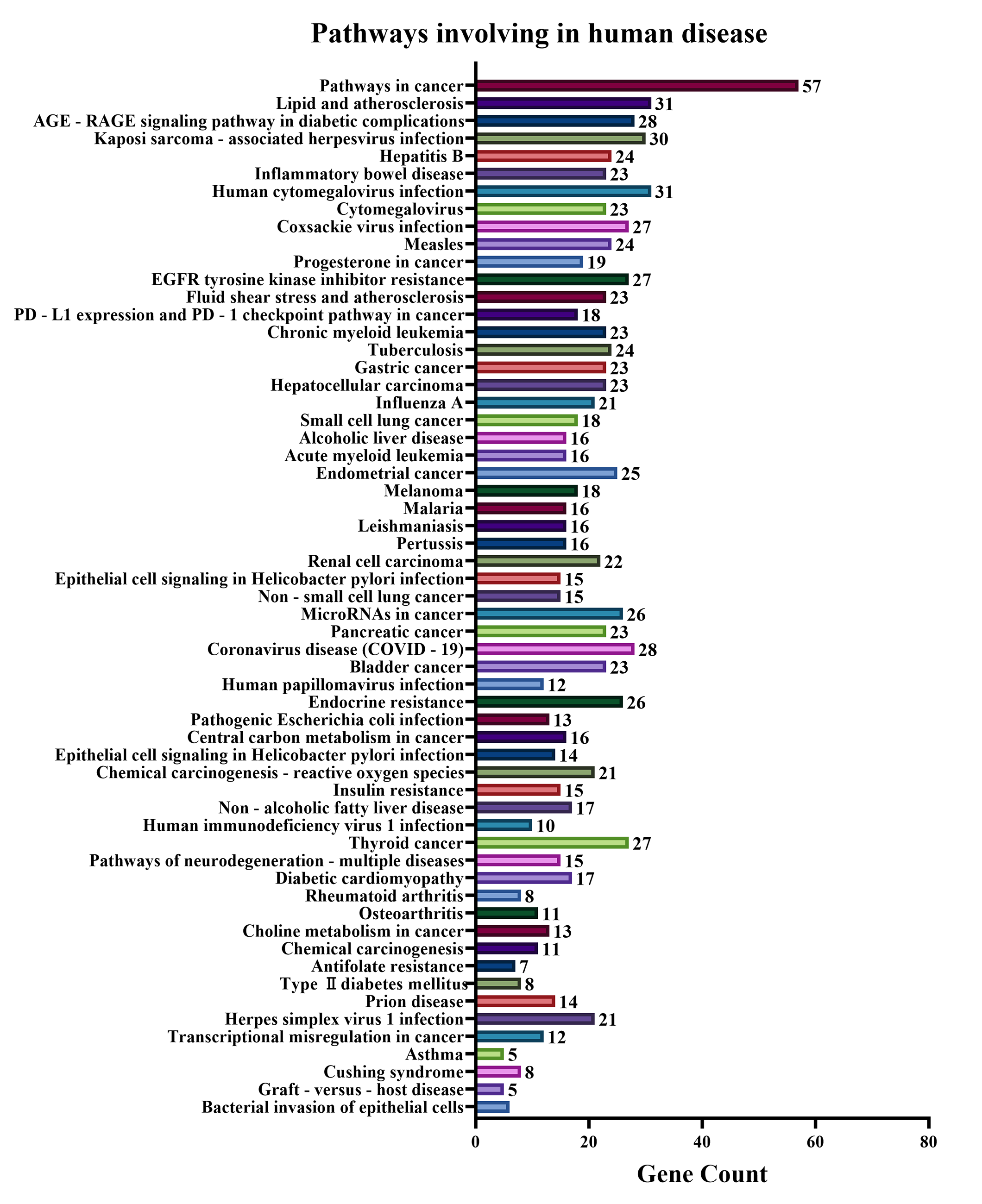

Supplement: S1 Fig — (TIF) [file pone.0335139.s002.tif]
